# Day-1 Competencies for Veterinarians Specific to Health Informatics

**DOI:** 10.3389/fvets.2021.651238

**Published:** 2021-06-11

**Authors:** Zenhwa Ben Ouyang, Jennifer Louise Hodgson, Elliot Robson, Kevin Havas, Elizabeth Stone, Zvonimir Poljak, Theresa Marie Bernardo

**Affiliations:** ^1^Department of Population Medicine, Ontario Veterinary College, University of Guelph, Guelph, ON, Canada; ^2^Department of Population Health Sciences, Virginia-Maryland College of Veterinary Medicine, Blacksburg, VA, United States; ^3^Eduworks, Corvallis, OR, United States; ^4^Department of Clinical Studies, University of Guelph, Guelph, ON, Canada

**Keywords:** competencies, veterinary education, health informatics, competency framework, information and communication technology, social media, health data

## Abstract

In 2015, the American Association of Veterinary Medical Colleges (AAVMC) developed the Competency-Based Veterinary Education (CBVE) framework to prepare practice-ready veterinarians through competency-based education, which is an outcomes-based approach to equipping students with the skills, knowledge, attitudes, values, and abilities to do their jobs. With increasing use of health informatics (HI: the use of information technology to deliver healthcare) by veterinarians, competencies in HI need to be developed. To reach consensus on a HI competency framework in this study, the Competency Framework Development (CFD) process was conducted using an online adaptation of Developing-A-Curriculum, an established methodology in veterinary medicine for reaching consensus among experts. The objectives of this study were to (1) create an HI competency framework for new veterinarians; (2) group the competency statements into common themes; (3) map the HI competency statements to the AAVMC competencies as illustrative sub-competencies; (4) provide insight into specific technologies that are currently relevant to new veterinary graduates; and (5) measure panelist satisfaction with the CFD process. The primary emphasis of the final HI competency framework was that veterinarians must be able to assess, select, and implement technology to optimize the client-patient experience, delivery of healthcare, and work-life balance for the veterinary team. Veterinarians must also continue their own education regarding technology by engaging relevant experts and opinion leaders.

## Introduction

In 2015 the American Association of Veterinary Medical Colleges (AAVMC) assembled representatives from 10 colleges in Canada, the Netherlands, the UK, and the USA to re-envision the veterinary curriculum and develop a common framework based on competencies (1). The framework, released in 2018, consisted of nine domains of competence and 32 competencies (1). This and other frameworks focused on veterinary education (2–4) can serve as a resource for colleges redesigning their curriculum as well as a roadmap for learners. Furthermore, student learning outcomes and competencies are evaluated as part of the accreditation process of the AVMA's Council on Education^1^, the Royal College of Veterinary Surgeons (RCVS)^2^, the Australasian Veterinary Boards Council (AVBC) (5) and others. The Competency-Based Veterinary Education (CBVE) framework was developed to train veterinary students to become “practice-ready” (1) and is described as “one of the most substantial pedagogical projects ever undertaken by the AAVMC” (1, p. i). To the authors' knowledge, an evaluation of the adoption and implementation of the competencies has not been conducted.

Competency-based education is a holistic (6) type of outcomes-based training for students or personnel who perform professional tasks requiring a combination of skills, knowledge, attitudes, personal values and abilities^3^ (7–10). Competency-based education considers cultural influences (6) and is, therefore, responsive to how society changes (7). It represents a shift from time-based to outcomes-based curriculum development (7).

In parallel with developments in medical education, calls for competency-based education in veterinary medicine began as early as 1989 and continue today in order to adapt to the changing landscape of veterinary practice (1, 2, 9, 11–15). The emergence of informatics is a significant recent change in the medical (16) and veterinary fields (17–24), and its relevance has been highlighted by the Coronavirus Disease 2019 (COVID-19) pandemic. Health informatics (HI) is a broad and rapidly evolving field, which is used here in general terms to describe the combination of health and information technology.

Medical schools began developing HI competency frameworks in the last decade. For example, the Certified Health Informatician Australasia Health Informatics Competencies Framework (25) drew on previous frameworks created by the Australian Health Informatics Education Council^4^, American Medical Informatics Association (26), International Medical Informatics Association (27) and Canada's Health Informatics Association^5^. In veterinary medicine, although general statements about HI may be included within competency frameworks, a framework that is focused specifically on HI for veterinarians may prove helpful.

In order to promote inclusion of HI in veterinary curricula, the objectives of this study were to: (1) develop a set of competencies relevant to new veterinarians regarding HI; (2) summarize the competencies by grouping the statements into common themes; (3) map the HI Competencies to the AAVMC competencies (1); (4) provide insight into specific technologies that are currently relevant to upcoming veterinarians; and (5) measure panelists' satisfaction with the methodology for competency development.

## Materials and Methods

### Background and Theory of Competency Framework Development

In this study, the technique of “competency-based framework development” (CFD)^3^ was used. This technique is based on an existing methodology, called “Developing-a-Curriculum” (DACUM) (28–30) that has been used in veterinary education (31, 32) as well as in other fields (29, 33) and utilizes an expert panel to reach a consensus on a competency framework. The main difference between CFD and DACUM is that, while DACUM requires in-person sessions, CFD is designed for the expert panel to convene online. This was an important advantage in the current study because it allowed for broad expertise without the travel time and expense required for in-person sessions.

In CFD, competency is defined as the application of knowledge and skills to do a job^3^. The principles inherent in CFD include: (1) The ideal number of expert panelists is between four and six; (2) Panelists must be practitioners of the job, rather than supervisors or educators of those practitioners; (3) The sum of competency statements developed comprises the competency framework; and (4) Each competency statement consists of underpinning knowledge, skills and/or abilities, as well as an example of how the competency could be assessed.

In competency frameworks produced using CFD, competencies are described using competency statements, which resemble learning objectives or learning outcomes and explicitly describe the tasks practitioners must perform. Further, CFD competency frameworks also contain the skills and knowledge underpinning each competency statement. Knowledge, as described under CFD, is “facts, principles, and beliefs” and can be shared with or acquired from others through communication, e.g., knowing to which authorities to report public health risks. A skill, as described under CFD, is the application of knowledge to complete a task and is attained or improved through training^3^. Under a classical veterinary educational context, an example of a skill may include being able to perform physical examinations and to create differential diagnoses. A parallel example of a skill for a veterinarian in health informatics could include performing a teletriage consultation.

Competencies are also observable and, therefore, assessable. In the CFD framework, assessments can occur at the level of the competency, as well as at the lower level of knowledge or skills necessary to achieve that competency. Assessing knowledge is typically done by recall or explanation. Assessment of a skill should be done by providing a situation during which a trainee can demonstrate the skill. A skill should not be assessed by recall or explanation. In CFD, assessment of competency is based on “indicators of competency,” which are positive evidence that someone can accomplish certain aspects of a competency^3^. For example, in a classical veterinary educational context, surgical trainees are required to be competent at performing surgery, however, only certain operations may be assessed (e.g., ovariohysterectomy). In a health informatics context, veterinary trainees may be required to be competent at performing remote consultations, however, only certain aspects of remote consultations may be assessed (e.g., teletriage).

### Overview of the Competency Framework Development Process

#### Selection of Panelists

Purposive sampling was used to identify six expert panelists who: (1) were veterinarians, (2) were in clinical practice within the previous year and (3) worked in one or more aspects of HI. We sought to maximize the mixture of skills and experiences within this small group of experts, e.g., telehealth, home visits, large animals, small animals, corporate experience. Of the six participants who were contacted; five participated in the study. Participants were sent an information letter and signed a consent letter describing the purpose of the study and the CFD process. The study was reviewed by the University of Guelph Research Ethics Board for compliance with federal guidelines for research involving humans (REB #: 17-10-037).

#### Facilitators

Two facilitators (primary and backup) from Eduworks^6^ (34) a company specializing in the CFD method^3^, facilitated the sessions with the expert panelists. The facilitators of the consensus sessions were experts in the CFD method and were not affiliated with the expert panelists or their supervisors or educators. One facilitator led the discussion while another facilitator recorded statements produced by the expert panel in real time. Prior to the first consensus session, panelists were provided with the whitepaper^3^ describing the CFD process. Four, 3-h working sessions, for a total of 12 h, were conducted online via Zoom^7^ between May 24, 2018 and June 14, 2018. Panelists could join the meeting from any location but were advised to select locations that were private and conducive to a meeting.

#### Consensus Sessions

At the beginning of the first session. the facilitator gave an orientation to the CFD process and asked the panelists “What do new practicing veterinarians need to know about health informatics?” Panelists were asked to discuss their understanding of HI without being given a specific definition and to contribute their concepts based on their own professional experiences. Panelists were then asked to brainstorm various tasks necessary for a new practicing veterinarian in HI. Draft competency statements were developed as the brainstorming proceeded and were visible to all participants on their screens via Google Sheets^8^ for immediate feedback and clarification. Once a statement was developed, it was refined until consensus was reached by all panelists. A similar process was undertaken for the knowledge, skills, abilities, and assessment statements. After all statements were completed, participants reviewed and provided feedback before the final session was over. The statements were then collated into a competency framework and sent out for final review to the expert panelists.

#### Competency Framework

The final document included the two main outputs of the CFD process: (1) a set of competency statements, each with accompanying statements of knowledge and skills; and (2) methods of assessment for each competency. From this point on, the competency statements and competency framework developed in the current study will be referred to as the “HI Competencies/Competency Statements” and “HI Competency Framework,” respectively.

### Thematic Analyses of the HI Competency Framework

Thematic analysis is a widely-used qualitative research method that identifies patterns, or themes, in data (e.g., literature, interviews, focus groups) (35). Thematic analyses were used to accomplish objectives two and four of this study. The thematic analyses consisted of the following steps outlined by Braune and Clark (35, 36): (1) “gain familiarity with the data”; (2) “generate initial codes”; (3) “search for themes”; (4) “review themes”; (5) “define and name themes” and (6) “produce the report.” In the current study, two thematic analyses were conducted to identify themes that were present through multiple competencies. In the first thematic analysis, themes were created based on the actions described in each competency statement. These resulting themes could be considered analogous to the Domains of Competence found in the CBVE Framework and helped facilitate the mapping of the HI competency statements to the CBVE competency statements (see below). A second thematic analysis was performed to identify specific technologies that were documented in the final HI Competency Framework to provide insight into relevant technologies for practicing veterinarians.

### Mapping HI Competencies to the CBVE Framework

The authors mapped the HI Competencies to those included in the CBVE Framework developed by the AAVMC Working Group on CBVE. “Competency,” as defined by the CBVE Framework, was “an observable ability of a health professional related to a specific activity that integrates knowledge, skills, values and attitudes” (1). The CBVE Framework also contained illustrative subcompetencies for each competency. It is important to note that the CBVE Framework was created for the totality of veterinary medicine, while the HI Framework was created for one aspect of veterinary medicine (HI). In this context, HI competency statements could be considered analogous to subcompetency statements in the CBVE Framework. Thus, HI competency statements were mapped to CBVE competency statements as subcompetencies.

### Measuring Panelist Satisfaction With the CFD Process

After completion of all of the consensus sessions, each panelist was asked to complete a 12-question survey (Table 1). The survey was designed to assess panelist satisfaction with the CFD methodology and process, the efficacy of the facilitator, and the efficacy of the collaboration tools (Google Sheets) and communication technology (Zoom) used during the consensus sessions.

**Table 1:** Survey to assess panelist satisfaction.

**1) Did the white paper on Competency Framework Development prepare you for the first session?**	**Frequency**
It prepared me well.	2
It provided some information, but not enough. I would have preferred more background.	1
It did not prepare me at all.	0
I don't think it's necessary to prepare participants for the first session.	2
**2-4) Please rate the facilitator's effectiveness for the following:**	
**2) Getting panelist participation**.	
0 (very negative)	0
1	0
2	0
3	1
4	1
5 (very positive)	1
**3) Guiding panelists in statement formation**.	
0 (very negative)	0
1	0
2	0
3	1
4	2
5 (very positive)	1
**4) Guiding panelists toward consensus**.	
0 (very negative)	0
1	0
2	0
3	1
4	2
5 (very positive)	1
**5) Competency framework development usually requires 12 h. For this project, the meetings were split into 4** **×** **3 h meetings. Please select how you would have preferred to meet. Select all that apply**.	
12 × 1 h meetings	0
6 × 2 h meetings	2
4 × 3 h meetings (no change)	1
3 × 4 h meetings	1
2 × 6 h meetings	1
1 × 12 h meeting	1
**6) What do you think would be the optimal group size for developing a competency framework?**	
1–3 panelists	0
4–6 panelists.	4
7 or more panelists.	0
**7) How did the fact that the meetings were virtual affect your willingness to attend?**	
It made me more willing to attend.	3
It had no influence on my willingness to attend.	0
It made me less willing to attend.	1
**8) How did the fact that the meetings were virtual impact your ability to attend?**	
It made it easier for me to attend.	4
It had no influence on whether I would attend.	0
It made it harder for me to attend.	0
**9) I would prefer meetings with:**	
Audio only.	0
Audio and optional video.	4
Audio and mandatory video.	0
**10) I think that if the meetings were in-person, the competency framework would have been:**	
More complete.	1
The same.	3
Less complete.	0
**11) Did you find Google Sheets to be an effective collaboration tool?**	
I found Google Sheets to be an effective tool for collaboration.	3
I found Google Sheets to be effective in some ways, but not in others.	1
I found Google Sheets to be ineffective.	0
**12) Do you agree with the final outcome of the competencies?**	
Yes.	4
No.	0

## Results

The eight HI Competency statements can be found in Table 2. An example of an HI competency that includes the underpinning knowledge, skills, and assessment statements, is provided in Table 3. The full competency framework can be found in Supplementary Table 1. A total of 8 competencies, 39 skills and 27 knowledge statements comprised the final HI Competency Framework.

**Table 2:** List of HI competency statements.

	**Competency statements**
1	The graduate actively seeks engagement and leadership within emerging technology in the non-veterinary animal health market.
2	The graduate advocates for effective use of current communication technology while respecting the privacy and regulatory implications on quality medical practice.
3	The graduate advocates the use of technology and innovation to facilitate quality practice management and improve work-life balance.
4	The graduate seeks opportunities to further their knowledge in data management, informatics, and communication technology.
5	The graduate selects appropriate communication technologies and manages their virtual footprint in a way that reflects well on the profession. The graduate navigates online controversies involving veterinary medicine in a professional manner and supports wellness of the profession.
6	The graduate uses medical and production software systems, and maintains records in a format that allows analysis and sharing.
7	The graduate utilizes technology to advance the surveillance and management of public health risks.
8	The new graduate utilizes data within an evidence based process to better promote animal health and welfare.

**Table 3:** HI competency statement 3 (including skills, knowledge and assessment statements).

**Competency statement**	**Skills, knowledge, assessment statements**
	Skills
The graduate advocates the use of technology and innovation to facilitate quality practice management and improve work-life balance. Description: The graduate is aware of situations in their practice environment that could be improved through the use of technology and/or data management practices. The graduate evaluates technologies that could save time and/or improve workflow.	Selects the right combination of internal and external technologies and services to streamline practice and improve work-life balance.
	Demonstrates the use of technological solutions to improve the efficiency of patient care, i.e., client communication, medical record keeping, follow up etc.
	Integrates external resources (technologies and services) to improve practice management and work-life balance.
	Advocates for technological solutions that enhance quality of practice and work-life balance.
	Evaluates emerging technologies and their impact on practice management and work-life balance.
	Knowledge
	The graduate recognizes that efficiency gained through the use of technology can have a positive impact on workplace productivity.
	The graduate recognizes where technology could improve practice and work-life balance.
	The graduate appreciates the importance of work-life balance.
	Assessment
	The graduate evaluates new medical record software that decreases the amount of time spent entering data.

During the first thematic analysis, a summary of the major themes for the HI competency statements were identified (Table 4). The three themes that emerged from the thematic analysis were Theme 1-Leadership; Theme 2-Continuing Education; and Theme 3-Technological Application. The Leadership theme was assigned to three HI competency statements (no. 1, 2, and 3) that used the words “leadership” or “advocacy” in the context of introducing technology to a veterinary community, e.g., veterinary clinic, or connecting the veterinary community with the technology community. The Continuing Education theme was assigned to the HI competency statement (no. 4) that described “furthering of knowledge.” The Technological Application theme was assigned to HI competency statements (no. 5, 6, 7, 8). that described “selecting technologies” or “using” or “maintaining” technologies.

**Table 4:** Thematic analysis of HI competency statements.

**Theme**	**Full competency statement**
**1 - Leadership**	
	The graduate actively seeks engagement and leadership within emerging technology in the non-veterinary animal health market.
	The graduate advocates for effective use of current communication technology while respecting the privacy and regulatory implications on quality medical practice.
	The graduate advocates the use of technology and innovation to facilitate quality practice management and improve work-life balance.
**2 - Continuing education**	
	The graduate seeks opportunities to further their knowledge in data management, informatics, and communication technology.
**3 - Technological application**	
	The graduate selects appropriate communication technologies and manages their virtual footprint in a way that reflects well on the profession. The graduate navigates online controversies involving veterinary medicine in a professional manner and supports wellness of the profession.
	The graduate uses medical and production software systems, and maintains records in a format that allows analysis and sharing.
	The graduate utilizes technology to advance the surveillance and management of public health risks.
	The new graduate utilizes data within an evidence based process to better promote animal health and welfare.

During the second thematic analysis, specific technologies were identified (Table 5). Four technology themes emerged, of which three referred to specific technologies: (1) social media and the internet (Tech-theme 1); (2) communication technology (Tech-theme 2); and (3) electronic records (Tech-theme 3). The fourth theme referred to data techniques (Tech-theme 4).

**Table 5:** Thematic analysis of technology themes.

**Tech-theme**	**Technology/technique**	**Examples**
1	Social media and internet	Social media, web presence, virtual footprint
2	Communication technology	Communication technology, telecommunication, telemedicine
3	Electronic records	Medical and production systems, medical records
4	Data	Data, data management, data analytics

Results of the panelist survey can be found in Table 1. Question 1 was the only question to be answered by all five panelists. Question 2 was answered by three panelists. All other questions, including the open-ended questions, were answered by four panelists. These four panelists will be referred to as the “remaining survey participants” for convenience. Panelists (all five) held mixed opinions as to how well the CFD whitepaper prepared them for the consensus sessions (Table 1, Question 1). The remaining survey panelists (with the exception of one for Question 2) gave a score of three or higher (out of five) for all categories of facilitator efficacy (Table 1, Questions 2–4). Although the remaining survey panelists were open to a variety of meeting frequencies and durations (Table 1, Question 5), none of the remaining survey panelists wanted to participate in 12 1-h sessions. Only one remaining survey panelist found the online format a deterrent to attending consensus sessions (Table 1, Questions 7–8). The remaining survey panelists were in agreement that video streaming should be optional for each participant. No remaining survey panelists disagreed with the final HI Competency Framework (Table 1, Question 12).

## Discussion

This study focused on five objectives to help promote the inclusion of HI into the veterinary curriculum.

### Objective 1: Develop Competencies Relevant to New Veterinarians Regarding Health Informatics

Veterinarians are facing a barrage of technologies all marketed as beneficial to the practice of veterinary medicine. For instance, the marketers of smart medical records, such as Vetspire^9^, and tools, like Suki^10^, that convert speech from clinical encounters into text, claim (37, 38) to streamline the documentation of cases and improve the accessibility of patient information through artificial intelligence. Wearable activity trackers, such as Whistle^11^ and FitBark^12^ for dogs and cats, Quantified Ag ear tags^13^ for cattle, and Smart Halter^14^, Equisense^15^ and Equinosis^16^ for horses, offer insights into the minute-by-minute health data of an animal, and some promise to monitor specific conditions such as pregnancy or lameness. Smart feeders, such as SureFeed^17^, PetSafe^18^, and PetNet^19^, provide measurable portions of food for pets to help in weight management and may even automatically order more food when supply is low. Robotic milkers^20^ are purported to promote the welfare of cows while improving production. These systems also collect and provide large amounts of data about each animal, as well as the herd, to the producer and veterinarian. Remote communication technologies have facilitated new models of healthcare delivery, such as LiveDVM^21^ and Fuzzy^22^, which incorporate aspects of telehealth. And finally, already-existing data in traditional veterinary records (paper or electronic) and diagnostic laboratory records may provide valuable insight into animal health. The expansion of technological solutions is especially evident in human medicine as large technology corporations have moved into healthcare. For example, Microsoft^23^ has partnered with Nuance Communications Inc.^24^ to develop an artificial intelligence-based ambient clinical documentation and decision support tool for physicians^25^, and Amazon has introduced Amazon Comprehend Medical^26^, an artificial intelligence-based natural language processing service designed to extract medical information from unstructured medical records.

As these technologies and others become more prevalent, veterinarians must have, or be able to acquire, the background necessary to critically assess these technologies before adoption or rejection. Veterinarians could accomplish this by, perhaps, assuming leadership positions that bridge the gap between veterinary professionals and the technology community (identified in Theme 1) (Table 4), or through continuing education throughout the veterinarian's career (identified in Theme 2) (Table 4). Veterinarians, when deciding on a technology, should consider whether the technology is appropriate for their practice, personnel, and clientele. In order to be useful, the technology should improve the delivery of healthcare to patients and improve the client-patient experience. Concurrently, the technology should streamline workflow and improve, or at least not worsen, work-life balance of hospital personnel. Further, veterinarians need to consider whether their practice, and personnel, possess the interest, willingness, expertise, and the resources to fully integrate a new technology into a practice and/or be able to identify and utilize a consultant to assist them e.g., Veterinary Integration Solutions^27^ (Theme 3: Technological application).

Decisions about whether to integrate new technologies into practice should be evidence-based when possible. Peer-reviewed literature is useful but may not contain studies about newer technologies. In order to assist future veterinarians to develop these skills, veterinary schools should consider integrating the use of relevant knowledge and skills regarding these technologies at appropriate points in the curriculum. There is a specific reference to information technology in the current CBVE framework, which is found under the “Professionalism and Professional Identity” domain of competence: (1): “Uses appropriate resources for learning and decision making (e.g., information technology, consultation with colleagues).” However, it is clear from the current study, that additional knowledge and skills regarding HI will be needed of our graduates in order for them to be appropriately prepared for these emerging technologies. We suggest that the HI competencies could serve as important subcompetencies in the CBVE framework that need to be explicitly identified so that veterinary students can recognize the relevance of technology. Without such training, veterinary students may choose to isolate themselves from technology instead of leveraging technologies when appropriate. As veterinarians, such attitudes could have negative impacts on their ability to deliver healthcare and attract clients. Veterinary students who see the benefits of technology but do not have this training may not be equipped to use these technologies to the fullest. Lastly, current veterinary students are likely the most technologically skilled generation of veterinary students since most of them are of the Millennial (39) and Gen Z (40) generations and tend to outpace older generations in adoption of technologies. Not providing these students with the resources necessary to navigate these technologies in clinical practice would be a missed opportunity.

### Objective 2: Summary of HI Competency Statements Into Common Themes

The thematic analysis presented in Table 4 show three underlying themes in the HI competency statements: leadership, continuing education, and technological application. A broad interpretation of HI competency statements grouped under the leadership theme is that graduates are able to bridge gaps between the established veterinary community and the tech community and/or the new veterinarian can introduce new HI technology into the veterinary profession. HI competency statements grouped under “continuing education” reflect the need for veterinarians to remain up-to-date in HI throughout their careers. And, HI competency statements grouped under “technological application” reflect that graduates must be able to select appropriate technologies and implement them into practice. These themes provide a paraphrase of what graduates may need to do in order to operate effectively in a HI world. For example, new graduates may be expected to be on the frontlines, deciding which technologies are accepted by the profession (Theme 1) as well as how they will best serve the profession (Theme 3). Veterinarians must remain educated (Theme 2) about these technologies and may rely on both conventional (coursework, continuing education, self-study) and non-conventional methods (networking with non-veterinary communities).

### Objective 3: Mapping HI Competency Statements to CBVE Competency Statements

Many of the current veterinary school curricula leave the onus of learning about HI to the student. Students may have personal experience with certain technologies; however, a curriculum that trains students to find opportunities to deepen their knowledge, to critically assess technologies and how to implement technologies would be synergistic with the attitude and technological proficiency of the current generation of students. There are many options for implementing aspects of the HI Competency Framework into a veterinary curriculum. Creating a set of courses that address technology is one possibility, however, veterinary school curricula is currently extremely dense with little room for additional coursework. Another strategy is to integrate technological considerations into existing competencies and courses. This study demonstrates that integration may be feasible since all HI competencies can be mapped to the CBVE Competency Framework (Table 6). For instance, the CBVE competency statement “Applies population management principles in compliance with legal regulations and economic realities” was mapped with the HI competency statement “The graduate uses medical and production software systems and maintains records in a format that allows analysis and sharing” (Table 6). An updated subcompetency statement for the CBVE competency statement based on the HI competency statement may be “Interfaces with technology to increase efficiency of collecting and assimilating patient information.” A learning session could include discussion on how wearable technology that owners buy at the pet store could provide valuable, potentially diagnostic, context, and data on the patient. Another example of a subcompetency based on this mapping is “Uses voice-to-text technology to improve the entry of data into the medical record to facilitate a more complete record while still being attentive to clients” (41–43). Given the constantly evolving nature of technology, any curricular development related to technology should not be static, or it would rapidly become out of date.

**Table 6:** Mapping of HI competency statements^a^ to CBVE competency statements.

**CBVE Framework**		**HI Competency Framework**
**Domains of competence**	**Competency statements**	**Competency statements**
**Animal population care and management**		
	Applies population management principles in compliance with legal regulations and economic realities	The graduate uses medical and production software systems and maintains records in a format that allows analysis and sharing (Statement 6).
		The new graduate utilizes data within an evidence-based process to better promote animal health and welfare (Statement 8).
**Public health**		
	Recognizes zoonotic diseases and responds accordingly	The graduate utilizes technology to advance the surveillance and management of public health risks (Statement 7).
	Promotes the health and safety of people and the environment	
**Communication**		
	Listens attentively and communicates professionally	The graduate advocates for effective use of current communication technology while respecting the privacy and regulatory implications on quality medical practice (Statement 2).
**Collaboration**		
	Solicits, respects, and integrates contributions from others	The graduate actively seeks engagement and leadership within emerging technology in the non-veterinary animal health market (Statement 1).
**Professionalism and professional identity**		
	Adopts an ethical approach to meeting professional obligations	The graduate selects appropriate communication technologies and manages their virtual footprint in a way that reflects well on the profession. The graduate navigates online controversies involving veterinary medicine in a professional manner and supports wellness of the profession (Statement 5).
	Practices time management	The graduate advocates the use of technology and innovation to facilitate quality practice management and improve work-life balance (Statement 3).
	Engages in self-directed learning and career planning	The graduate seeks opportunities to further their knowledge in data management, informatics, and communication technology (Statement 4).

a *The HI competency statements may be viewed as “illustrative subcompetencies” in the CBVE Framework*.

This study provides evidence that online consensus methods, such as CFD, may be a viable methodology to update competencies on HI. This process required a significant input of time and resources, although not on the same scale as the current CBVE. Both were written in a manner to remain relevant over time. With regard to more minor, and frequently encountered, updates to technology, however, the challenge will be to create a means of dynamically updating learning opportunities (which could be co-created with students) in a manner that serves as a springboard for autodidacticism. In order to equip students to continue this trajectory after graduation and to compensate for any potential “petrification” [as described in dental (44) and medical (45) education] of the veterinary curriculum (46), it is also necessary to teach them to tap into other sources of expertise, such as experienced colleagues and key opinion leaders in HI. For those veterinarians who seek greater engagement, it is critical to build professional networks that include experienced individuals outside of veterinary medicine, including, for example, experts in human health technology and engineering. The American Medical Informatics Association^28^ may be a good resource for developing such connections. Veterinarians may become directly involved with technology companies through employment or by serving as consultants or board members. This may be especially useful to technology companies that are driven by individuals with expertise other than veterinary medicine or animal health as the veterinarian can provide valuable guidance on how to improve delivery of healthcare, client-patient experience and veterinarian work-life balance. Further, these veterinarians may become valuable sources of information to other veterinarians regarding technology. Veterinarians may seek relevant educational opportunities at conferences and workshops (e.g., the Association for Veterinary^29^ Informatics provides continuing education opportunities in addition to professional networking) or they may seek advanced training and degrees in programs within veterinary medicine or beyond that investigate development and application of new HI. Another approach is to identify and follow key opinion leaders on social media. Ultimately, professional networks may be the most effective method of learning and staying up-to-date with the technological landscape of veterinary medicine.

Note that the panelists in the current study referred to the student as “graduate,” which reflects the fact that all expert panelists were practitioners first, rather than academic educators. This type of expert panel provides a prospective approach to curriculum development and focuses heavily on what graduates will encounter and need to do in practice after graduation and how to prepare for it. In contrast, the CBVE Framework is more focused on the student experience and knowledge and skills acquisition while still in school. Although the HI Competency Framework can be integrated into an existing competency framework, such as the CBVE Framework, it may be useful to be able to view the HI Competency Framework as a separate entity if the goal is to develop curricula specific to HI. Ultimately, the HI Competency Framework is not at odds with other frameworks. Rather, it is meant to complement and augment aspects of pre-existing veterinary competency frameworks relevant to health informatics.

### Objective 4: Specific Technologies Relevant to Upcoming Graduates of Veterinary Medicine

The internet and social media (Tech-theme 1) (Table 5) and communication technologies (Tech-theme2) (Table 5) have changed the way veterinarians and clients interact. Clients often rely on the internet and social media to find a veterinarian. Clients may not only have their first conversation with personnel from a veterinary practice through these platforms but may also book appointments through these websites and social media. Communication technologies including cell phones, text and instant messaging, live video chats, and emails have the potential to improve the ways veterinarians and clients converse and transfer relevant clinical information. However, in order to communicate appropriately using these technologies, the veterinarian must first understand their clientele and how they might use, or not use, these technologies. For example, as has been emphasized during the COVID-19 pandemic, these technologies may not be uniformly available across the entire clientele or potential new clients. Additionally, even when technologies are available, they may not be successfully adopted by clients or used for their intended purpose. Clients may also choose veterinary hospitals based on whether they can interact with their veterinarians through certain technologies. Veterinarians must then decide what is the best way to reach new clients and communicate and transfer information with current clients. Furthermore, if a veterinarian chooses to maintain a presence on the internet and/or social media, they must conduct themselves in ways that reflect positively on themselves and the profession (Table 2, HI Competency 5).

Many of the technologies listed above collect a variety of data ranging from animal activity (e.g., wearables) to hospital or farm financial data (e.g., medical or production records) (Tech-theme 3) (Table 5). With the “internet-of-things,” datasets can be combined to form larger, more complex and varied datasets than those typically seen in veterinary medicine and may require non-traditional means of analysis, e.g., machine learning, predictive analytics (Tech-theme 4) (Table 5). Although veterinarians may not be involved with every stage of knowledge translation, they should minimally understand the importance of data collection in expanding knowledge in veterinary medicine. For example, veterinarians may have minimal involvement with data analysis, but may have greater involvement with interpretation of results and application in the clinical setting. This is especially true with technologies that depend on the veterinarian for data collection, e.g., electronic medical records. In this case, the veterinarian should understand that effective and accurate data entry can improve data quality and streamline research. This may require the veterinarian to be familiar with standardized terminology, such as the Veterinary Extension of SNOMED CT, which includes problems and diagnosis terms developed for small animals^30^ (AAHA—American Animal Hospital Association), horses^31^ (AAEP—American Association of Equine Practitioners) and small animal specialties^32^ (SAS). Veterinarians should also be aware of, and adept at navigating controversies surrounding data, e.g., privacy, security, and data ownership.

It is important to note that veterinarians may not work directly with many of these technologies, e.g., wearables, smart feeders, robotic milkers, and may only encounter them when a client brings it to their attention. Such technologies may be owned by clients and may provide large amounts of clinical data that can be transferred through communication technologies (e.g., email). Thus, the veterinarian may be presented with large amounts of information from devices that are directly available to clients and for which the veterinarian received little training. The information may be presented in formats unfamiliar to the veterinarian, and the veterinarian may not fully understand the technology that captured the data (e.g., efficacy, accuracy, reliability). Networking with the emerging technology community (Theme 1: Leadership) and seeking opportunities for continuing education (Theme 2: Continuing education) are good ways to stay updated on and remain vigilant of new technologies. Doing so will also allow veterinarians to address questions about these technologies with clients and to improve the delivery of healthcare (Theme 3: Technological application).

Many innovations in veterinary technology are driven by individuals outside of veterinary medicine or animal health. Veterinarian involvement with technology development at an early stage could be highly effective in guiding innovation toward the goals of improving healthcare delivery to pets, client-patient experience, and veterinary work-life balance. These veterinarians may also serve as valuable sources of expertise on technologies that were developed or are in the process of being developed by these companies. However, the onus will be on the veterinarian to become familiar with the company's business environment and what drives company decisions.

### Objective 5: Panelist Satisfaction With CFD Methodology

Under the current context of COVID-19, online collaborative work has become extremely relevant. Although the panel for the present study was convened before the pandemic, the adaptation of CFD, as a modification of the DACUM occupational analysis technique, is a viable method to reach consensus during a pandemic or other circumstances when face-to-face meetings are not possible or desirable. Another advantage of the CFD method is lower costs in convening the expert panel as well as increased convenience for panelists to participate (though not necessarily willingness) (Table 1). Panelists in the current study, however, largely agreed with the outcome of the process (Table 1). These results could reflect a skillful facilitator and/or an enthusiastic expert panel who were already experienced and supportive of technology.

### Limitations

There are a number of limitations to this study. The panel consisted of practitioners with a mixture of small animal, large animal, corporate, and start-up experience. Given the large degree of variation in both veterinary practice and technology, not all perspectives on what new veterinarians need to know regarding HI may have been considered by the five panelists. We tried to address this by selecting expert panelists from as many sectors of the industry as possible while still adhering to the recommended range of four to seven participants for development of CFD frameworks.

The CBVE Framework and HI Competency Framework were created using different methodologies. Thus, there was not complete congruence between the competency statements of the two frameworks. Furthermore, the frameworks were created for different purposes. The CBVE Framework was designed for the totality of veterinary education while the HI Competency Framework focused specifically on HI. It was ultimately decided that mapping the HI competency statements to the CBVE subcompetency statements was the best fit. The final mapping presented here is one interpretation, but provides an example of how competencies in HI could be included in an existing framework and curriculum.

Another limitation is that the HI Competency Framework was created within a certain temporal context of veterinary medicine and technology. The implications of the HI Competency Framework could be affected by changes in veterinary medicine and technology. However, the HI Competency Framework was created to remain relevant with the rapidly changing environment of technology, i.e., panelists attempted to create competencies that would be relevant even when technology changed. This may help keep the HI Competency Framework relevant through time.

Although we assessed panelist satisfaction of the CFD methodology (Table 1), the results reflected the opinions of at most five panelists. Panelists were also limited to the profession of veterinary medicine with experience in HI. Thus, generalizations may not be appropriate. However, the assessment of panelist satisfaction did help us understand whether panelists agreed with the outcome, providing some validation. Further, it provided some insight into how the panelists experienced the CFD method. Such information may be useful when running future CFD sessions with a similar group of expert panelists.

## Conclusions

This study provides a practical, expert perspective on what new veterinarians need to know about HI based on a consensus of practicing veterinarians. New veterinarians must be able to assess, select, and implement technology with consideration to the client-patient experience, optimal delivery of healthcare, and work-life balance for the veterinary team. Veterinarians must also be able to continue their own education regarding technology by engaging with relevant experts and key opinion leaders. Updates to HI competencies due to significant new disruptions in technologies can be addressed via the methodologies that convene experts through communication technologies, such as CFD. This may be an important consideration for expert groups that are difficult to convene in-person or during times where in-person sessions are not possible. In this manner, the competencies in the current HI framework could be adapted to accommodate changes in HI and continue to be incorporated into veterinary curricula. The current generation of students, many of whom are technologically savvy, may not only be especially receptive to such training, but may also be able to assist instructors by helping to update the curriculum.

## Data Availability Statement

The original contributions presented in the study are included in the article/Supplementary Material, further inquiries can be directed to the corresponding author/s.

## Ethics Statement

The studies involving human participants were reviewed and approved by University of Guelph Research Ethics Board. The patients/participants provided their written informed consent to participate in this study.

## Author Contributions

ZO and TB with help from ZP and ES conceived and led the project. ZO and TB were responsible for securing partnerships with Eduworks. ZO and TB provided relevant background for ER and KH. ZO and TB also sought and identified expert panelists. ZO and TB with guidance from ZP and ES completed the Research Ethics Board application. ZO, ES, ZP, and TB developed the survey for the expert panelists. ES, ZP, and TB assisted ZO with thematic analyses. ZO with guidance from ES, ZP, and TB wrote the manuscript. All authors contributed to manuscript revision.

## Conflict of Interest

The authors declare that the research was conducted in the absence of any commercial or financial relationships that could be construed as a potential conflict of interest.

## Footnotes

^1^American Veterinary Medical Association. (2018). *Accreditation Policies and Procedures of the AVMA Council on Education (COE)*. https://www.avma.org/ProfessionalDevelopment/Education/Accreditation/Colleges/Pages/coe-pp.aspx (accessed November 5, 2019).

^2^Royal College of Veterinary Surgeons. (2020). *Accrediting veterinary degrees*. https://www.rcvs.org.uk/setting-standards/accrediting-primary-qualifications/accrediting-veterinary-degrees/ (accessed September 15, 2020).

^3^*Eduworks Competency and Skills Management*. (n.d.). https://www.eduworks.com/competency-skills-mgmt.html (December 10, 2019).

^4^*Health informatics: scope, careers and competencies*. (2011). https://www.ahiec.org.au/docs/AHIEC_HI_Scope_Careers_and_Competencies_V1-9.pdf. (accessed November 5, 2019).

^5^*Health Informatics Professional Core Competencies*. (2012). https://digitalhealthcanada.com/wp-content/uploads/2019/07/Health-Informatics-Core-Competencies.pdf. (accessed November 5, 2019).

^6^*Eduworks*. (n.d.). Retrieved September 14, 2020, from https://eduworks.com/.

^7^Zoom Video Communications Inc. (2016). *Security Guide:* Zoom Video Communications Inc.

^8^*Google Sheets: Free Online Spreadsheets for Personal Use*. (n.d.). https://www.google.ca/sheets/about/. (accessed December 10, 2019).

^9^*Vetspire | AI-driven Veterinary Practice Management Software*. (n.d.). https://vetspire.com/. (accessed December 10, 2019).

^10^*Suki*. (2020). https://www.suki.ai/. (accessed December 10, 2019).

^11^*Whistle GO* & *GO Explore GPS Pet Trackers*. (n.d.). https://www.whistle.com/. (accessed December 10, 2019).

^12^*FitBark GPS* & *Activity Monitor | Healthy Together*. (n.d.). https://www.fitbark.com/ (accessed December 10, 2019).

^13^*Quantified AG® | The Best Cattle Ear Tag To Identify Cattle Illness*. (n.d.). https://quantifiedag.com/ (accessed December 10, 2019].

^14^*Smart Halter*^*TM*^ *| Early Warning Detection for Horses – NIGHTWATCH*. (n.d.). https://www.smarthalter.com/ (accessed December 10, 2019).

^15^*Equisense Shop*. (n.d.). https://equisense.com/ (accessed December 10, 2019).

^16^*Equinosis | Lameness Locator : Precision Lameness Measurement*. (n.d.). https://equinosis.com/. (accessed December 10, 2019).

^17^*Pet Feeding Solutions*. (n.d.). https://www.surepetcare.com/en-ca/pet-feeder (accessed December 10, 2019).

^18^*PetSafe® Official Website*. (n.d.). https://www.petsafe.net/ (accessed December 10, 2019).

^19^*petnet.io*. (n.d.). https://www.petnet.io/ (accessed December 10, 2019).

^20^*Lely Canada - Lely*. (n.d.). https://www.lely.com/ca/en/ (accessed December 10, 2019).

^21^Moser, S. A. (n.d.). New veterinary telemedicine service aims to save clients time, money. *Dvm360.Com* (accessed December 10, 2020).

^22^*Fuzzy - The best in-home veterinary care*. (n.d.). https://www.yourfuzzy.com/ (accessed December 10, 2019).

^23^*Microsoft - Official Home Page*. (n.d.). https://www.microsoft.com/en-ca/ (accessed December 10, 2019).

^24^*Nuance - Conversational AI for Healthcare and Customer Engagement | Nuance*. (n.d.). Retrieved from: https://www.nuance.com/index.html (accessed December 10, 2019).

^25^*Nuance and Microsoft partner to transform the doctor-patient experience - Stories*. (n.d.). https://news.microsoft.com/2019/10/17/nuance-and-microsoft-partner-to-transform-the-doctor-patient-experience/ (accessed December 10, 2019).

^26^*Amazon Comprehend Medical*. (n.d.). https://aws.amazon.com/comprehend/medical/ (accessed December 10, 2019).

^27^*Veterinary Integration Solutions – Consolidator Operating Platform*. (n.d.). Retrieved September 14, 2020, from https://vetintegrations.com/.

^28^*AMIA | Informatics Professionals. Leading the Way*. (n.d.). Retrieved April 15, 2021, from https://www.amia.org/.

^29^*Association for Veterinary Informatics*. (n.d.). Retrieved April 15, 2021, from https://www.avinformatics.org/.

^30^*AAHA Problem and Diagnosis Terms*. (n.d.). Retrieved April 15, 2021, from https://www.aaha.org/practice-resources/running-your-practice/diagnostic-terms/.

^31^*Developing a SNOMED-CT® based diagnostic terms list for the American Association of Equine Practitioners (AAEP)*. (n.d.). Retrieved April 15, 2021, from https://vtsl.vetmed.vt.edu/AAEP//.

^32^*Small Animal Specialty Problem* & *Diagnosis Terms*. (n.d.). Retrieved April 15, 2021, from https://vtsl.vetmed.vt.edu/SAS/homepage.cfm.
