# Supplementary material for: Day-1 Competencies for Veterinarians Specific to Health Informatics
Source: Front Vet Sci. 2021 Jun 11;8:651238. doi: 10.3389/fvets.2021.651238 (PMC8231916; doi:10.3389/fvets.2021.651238)
Supplement: Supplementary file 1 [file Table_1.DOCX]

**Supplementary material**

Supplementary table 1. Full Health Informatics Competency Framework.

| **Competency** | **Skills, knowledge, abilities** |
| --- | --- |
| **HI Competency 1:**  The graduate actively seeks engagement and leadership within emerging technology in the non-veterinary animal health market.  Description: The graduate actively seeks leadership roles within emerging models of animal healthcare. The graduate uses their veterinary knowledge to help strengthen and enhance the human-animal bond, advance animal husbandry, and ensure the health and wellness of all animals. | Skills (5)   - Approaches new technologies with an evidence-based methodology. - Reports emerging animal health innovation that inappropriately offers veterinary health services. - Maintains awareness of the changing landscape within the animal health community. - Ethically promotes the voice of the veterinarian in the animal health community. - Seeks involvement with the animal startup community.   Knowledge, abilities (4)   - The graduate is aware of emerging technologies and their potential impact on the veterinary profession. - The graduate believes veterinarians should be actively involved in emerging technologies in the non veterinary animal health market. - The graduate has a strong degree of respect for the human animal bond and how technology could impact that bond. - The graduate is driven to be involved in emerging non veterinary animal health technologies.   Assessment: Outside of their day-to-day practice responsibilities, the graduate is actively involved with animal health innovation external to the veterinary community. Example: The graduate consults or serves as a board member of newly formed pet-consumer startup companies. |
| **HI Competency 2:**  The graduate advocates for effective use of current communication technology while respecting the privacy and regulatory implications on quality medical practice.  Description: The graduate is aware of current communication technologies and how they could impact the clinic's ability to promote animal well being. The graduate is knowledgeable in current regulatory and privacy law and how they impact the use of technology. The graduate can effectively communicate to the practice how technology can improve client experience, facilitate case management, strengthen continuity of care, and improve work-life balance. | Skills (6)   - Respects evolving regulation relating to telemedicine. - Promotes the use of telecommunication technology to improve the client and patient experience. - Improves triage of clinical cases by using relevant communication technology. - Improves case management and continuity of care by using telecommunication technology. - Utilizes technology with a focus on improving the client and patient experience. - Retrieves and reviews medical data using telehealth technology ahead of physical appointments.   Knowledge, abilities (5)   - Promotes the use of telecommunication technology to improve the client and patient experience. - The graduate recognizes the limitations in the application of new technologies. - The graduate is aware of how data is stored regarding communication technology. - The graduate believes communication technology can improve practice, client experience, work-life balance, and animal welfare. - The graduate is aware of current communication technologies.   Assessment: The graduate promotes adoption of a new communication technology to a practice. Remains current with local regulatory, and privacy, legislation |
| **HI Competency 3:**  The graduate advocates the use of technology and innovation to facilitate quality practice management and improve work-life balance.  Description: The graduate is aware of situations in their practice environment that could be improved through the use of technology and/or data management practices. The graduate evaluates technologies that could save time and/or improve workflow. | Skills (5)   - Selects the right combination of internal and external technologies and services to streamline practice and improve work-life balance. - Demonstrates the use of technological solutions to improve the efficiency of patient care, i.e., client communication, medical record keeping, follow up etc. - Integrates external resources (technologies and services) to improve practice management and work-life balance. - Advocates for technological solutions that enhance quality of practice and work-life balance. - Evaluates emerging technologies and their impact on practice management and work-life balance.   Knowledge, abilities (3)   - The graduate recognizes that efficiency gained through the use of technology can have a positive impact on workplace productivity. - The graduate recognizes where technology could improve practice and work-life balance. - The graduate appreciates the importance of work-life balance.   Assessment: The graduate evaluates new medical record software that decreases the amount of time spent entering data. |
| **HI Competency 4:**  The graduate seeks opportunities to further their knowledge in data management, informatics, and communication technology.  Description: The graduate devotes a reasonable effort to exploring, critically evaluating, and understanding new methods of data management, informatics, and emerging trends in communication technology. The graduate actively learns new technology and processes with respect to informatics and promotes new ideas and practices with their peer group. | Skills (4)   - Participates in relevant conferences and webinars to learn new skills and processes or stay current on emerging technologies. - Joins and maintains an active participation in relevant professional networking groups. - Evaluates, shares, and discusses emerging technologies with peers and colleagues. - Searches literature in a critical fashion to retrieve relevant journals and information.   Knowledge, abilities (3)   - The graduate believes in continuing education in data management, informatics, and communication technology. - The graduate has a passion for advanced data management and information technology. - The graduate finds quality continuing education resources (courses, classes, webinars, etc.).   Assessment: The new graduate enrolls themself in a social media data analytics course. |
| **HI Competency 5:**  The graduate selects appropriate communication technologies and manages their virtual footprint in a way that reflects well on the profession. The graduate navigates online controversies involving veterinary medicine in a professional manner and supports wellness of the profession.  Description: The graduate evaluates communication technologies and tools to ensure they are professional. The graduate communicates effectively using these tools in a way that reflects well on themselves and the veterinary profession. The graduate's communications respect privacy law and uphold professionalism. | Skills (6)   - Contributes to the public's knowledge and awareness of pertinent veterinary issues. - Applies secure and effective communication technology. - Evaluates communication technologies and tools to ensure they are professional. - Manages social media and web presence in a way that reflects core veterinary values. - Upholds privacy law related to communication technology. - Can identify privacy risks of communication technologies.   Knowledge, abilities (2)   - The graduate applies privacy law and regulatory law regarding telemedicine. - The graduate believes it is important to maintain a professional image when using technology.   Assessment: The graduate uses appropriate technologies to carry themselves in a professional manner. |
| **HI Competency 6:**  The graduate leverages medical and production software systems, and maintains records in a format that allows analysis and sharing.  Description: The graduate is aware of key aspects of medical record systems and the importance of maintaining medical records in an accurate and consistent format. The graduate evaluates the medical record content and systems with consideration to the potential for medical data sharing and analysis internally and externally. | Skill (3)   - Maintains awareness of current and emerging regulatory requirements. - Monitors emerging technologies and trends in medical and production software systems. - Inputs medical, production, and other data in a format that allows for extraction and analysis.   Knowledge, abilities (3)   - The graduate inputs data in a way that allows for analysis. - The graduate extracts data from medical record and production software systems. - The graduate believes that improved data analysis will be essential to the advancement of knowledge within and outside the profession.   Assessment: The graduate keeps medical records in a format that is consistent and practical for larger scale analysis. |
| **HI Competency 7:**  The graduate utilizes technology to advance the surveillance and management of public health risks.  Description: The graduate reports and informs authorities, peers, and clients of public health risks. The graduate is aware of reporting and surveillance platforms and stays current with both at local, regional, national, and global levels. The graduate understands the importance of reporting potential risks and diseases that could affect the public and how they could protect animals and people by sharing information. The graduate is willing to contribute new ideas to help advance the practice of surveillance and public health risk management. | Skills (6)   - Organizes disease outbreak data using formats that facilitate collection and analysis by authorities. - Utilizes communication systems that allow for better educating of public risks to clients. - Engages clients to report diseases to aggregate better data. - Maintains awareness of government reporting processes and platforms. - Promotes new methods of surveillance and management that advance public health. - Reports public health risks using appropriate reporting channels.   Knowledge, abilities (4)   - The graduate recalls the various surveillance platforms at a local, regional, and national level. - The graduate believes in sharing information and disseminating public health risks to peers, clients, and the public. - The graduate demonstrates they are up-to-date on reportable diseases. - The graduate specifies which authorities to report public health risks.   Assessment: The graduate leverages current communication technology to inform their clients and the public of public health concerns in their community. |
| **HI Competency 8:**  The new graduate utilizes data within an evidence based process to better promote animal health and welfare.  Description: The new graduate utilizes current or emerging technology to analyze data in the promotion of evidence-based medicine, improving animal health and welfare decisions. | Skills (4)   - Iterates to improve work processes and case management based on evidence and feedback. - Incorporates responses (clicks, ratings, views, etc.) as constructive feedback. - Leverages appropriate technology for a particular purpose. (Ex: Animal-friendly handling practices video on Youtube.) - Analyzes data to enhance decisions pertaining to animal health and welfare.   Knowledge, abilities (3)   - The graduate believes that continuous advancement in data analytics can improve health and animal welfare decisions. - The graduate finds evidence-based data. - The graduate utilizes data to promote animal health and welfare.   Assessment: The graduate reviews existing electronic medical records to improve medical decision making for case management. |
